# Correction: The internet of things deployed for occupational health and safety purposes: A qualitative study of opportunities and ethical issues

**DOI:** 10.1371/journal.pone.0331742

**Published:** 2025-09-04

**Authors:** Maeva El Bouchikhi, Sophie Weerts, Christine Clavien

The first author’s initials appear incorrectly in the citation. The correct citation is: El Bouchikhi M, Weerts S, Clavien C (2024) The internet of things deployed for occupational health and safety purposes: A qualitative study of opportunities and ethical issues. PLoS ONE 19(12): e0315671. https://doi.org/10.1371/journal.pone.0315671.
