# Correction: The internet of things deployed for occupational health and safety purposes: A qualitative study of opportunities and ethical issues

**DOI:** 10.1371/journal.pone.0335283

**Published:** 2025-10-22

**Authors:** The PLOS One Staff

## Notice of Republication

This article was republished on October 9, 2025, to correct errors in the first author’s initials appearing incorrectly in the citation. The correct citation is: El Bouchikhi M, Weerts S, Clavien C (2024) The internet of things deployed for occupational health and safety purposes: A qualitative study of opportunities and ethical issues. PLoS ONE 19(12): e0315671. https://doi.org/10.1371/journal.pone.0315671.

## Supporting information

S1 File Originally published, uncorrected article. (PDF)

S2 File Republished, corrected article. (PDF)
