# Supplementary material for: The internet of things deployed for occupational health and safety purposes: A qualitative study of opportunities and ethical issues
Source: PLoS One. 2024 Dec 17;19(12):e0315671. doi: 10.1371/journal.pone.0315671 (PMC11651608; doi:10.1371/journal.pone.0315671)
Supplement: S1 Text — (DOCX) [file pone.0315671.s001.docx]

# S1 Text. Interview grids

We constructed our interview grid in English and French (all our coders are bilingual), with the aim of capturing a broad range of perspectives on the benefits, challenges, and risks associated with IoT devices for occupational health that could plausibly or that were already used in Switzerland. For this, we developed three fictional but plausible scenarios (see our interview grids below) of types of devices that are already being used across the globe: the deployment of connected chairs in the workplace to help employees avoid and correct bad posture (Posture-tracker Scenario); the use of smart-watches in the context of a corporate wellness program designed to motivate employees to make more daily steps (Step-tracker Scenario); the deployment of sound trackers in employees’ offices and homes to detect and reduce stress level (Sound-trackers Scenario). In order to cover as many relevant situations as possible, our three scenarios diverged on the following parameters: type of device used (more or less physically perceptible, more or less used in everyday-life), health purpose of the device (to prevent physical *versus* mental illness), time of implementation of the device (deployed over a short *versus* long period in the workplace), mode of decision for implementing the device (after collective consultation with employees, after consultation with employees representatives, without consultation), extension of activity of the device (workplace only *versus* extension in employee’s private sphere), type of data collected (postural data, personal data, health data, behavioural data, speech tone, etc.), type of data storage (in-house *versus* outsourced to the device’s provider), mode of data analysis (done in-house *versus* outsourced to the device’s provider), data accessibility (to employees only *versus* to physician or Human Resources [HR] or direct managers). To confirm that our scenarios were realistic, and to obtain a first gut feeling appreciation of participants, the interview grid included three starting closed questions: “Could this scenario be realistically used in Switzerland?”; “Do you know Swiss companies using similar IoT technology (provide a yes/no answer – no need to tell names) And how many companies?”; “Could this scenario be legally used in Switzerland ?” (the latter question was added in the course of the study, thus we do not have answers from all participants); “Would you (as employer versus as employee) be in favour of implementing this solution?”. We then proceed with an open question: “In your view, what are the chances (opportunities, advantages) and the ethical issues (possible risks, topic of controversy) related to this scenario?”. In addition, we developed follow-up questions to be used in case participants did not spontaneously discuss some issues that we considered relevant, in particular issues related to data flow: for each scenario, we showed to participants a map of the data flow process (figures in the interview grids below) and asked whether they saw new or further issues related to that process. We also asked additional questions on variations on the scenarios, such as “What if the data analysis was done in-house by the HR unit of the company?” (Posture-tracker Scen & Sound-trakers Scen) or “What if the individual data reports were transmitted to the occupational physician or to the HR service?” (Step-tracker Scen). In the sound-tracker scenario, we asked participants for their views on the role of the local manager, and “What if the computers are also equipped with a camera for capturing facial expressions and eye movement?” At the end of the interview, we added a global follow-up question on the decisions procedure ahead of the deployment of the device, and the role employees should take in that process: should employees or their representatives be consulted or can the decision be taken in a top-down manner?

| **Scenario I - Posture-tracker**  The company CHEERS decides to replace existing furniture in its main production hall because employees regularly reported back pain. In the past years, the in-house occupational physician has diagnosed several chronic illnesses due to employees’ posture while working on repetitive tasks.  Three types of new chairs are pre-selected, including a classic and a more high-tech option. After a collective consultation with the occupational physician and the employees working in that production hall, the heads of the company decide to buy the high-tech set of furniture that was preferred by most employees.  These smart chairs are produced by the company FitChair. The chairs detect incorrect neck, head and back posture and a red light switches on whenever its user remains in the incorrect posture over several minutes. They also produce a low sound to inform users when they remain seated for too long. FitChairs are delivered with a program that enables storage of data (collected by the chairs) about users’ posture and sitting time. Light and sound can be activated or deactivated by the employees as they wish. These data are stored on CHEERS’ cloud storage. They are periodically sent to BioData, a company specialised in analysing data from biosensor devices. BioData produces individual reports including health advice and sends them directly to the employees. BioData also produces general anonymised reports and sends them to CHEERS’ occupational physician. These reports are used for long-term evaluations of employees’ working conditions.  **Discussion Grid**  We will have two kinds of question. I’ll start with quick questions requiring very short one-sentence answers. After that, we will take more time to address more open questions.  *Additional instructions during the focus group: When answering this open question, please keep in mind that it is important to respect some rules guaranteeing an ethical discussion. First, everyone should have the opportunity to express his/her view. It means that if somebody takes too much space in the discussion, we may remind him/her of this rule. So please do not monopolize the floor. Second rule: different views are most welcome. Remember that the aim of the workshop is to uncover all kinds of issues, not to resolve a particular debate or to make everybody agree on one solution. So do not try to convince the others (this can be done in the coffee break). Third: it is important at all times to maintain a respectful attitude towards each other. Try to be inclusive, and respect the opinions independently of participants’ position, gender, etc.*  **Closed questions:**   - Could this scenario realistically be used in Switzerland? Please respond in one sentence.   - If no, why? (in one to two sentences)   - If yes, do you know Swiss companies using similar IoT technology? Please, provide a yes/no answer and no need to tell names. How many companies? - Could this scenario be legally acceptable in Switzerland? Yes or no? - Would you, as an employee / head of the company CHEERS, be in favour of buying FitChairs? Please respond in one sentence.   **Main open question:**   - In your view, what are the chances (opportunities, advantages) and the ethical issues (possible risks, topic of controversy) related to this scenario?   **Follow up questions:**   - Here is a figure illustrating the data flow in this scenario (see Figure below). Do further issues come to your mind when looking at the data flow process? - What if the analytics (data analysis) is done in-house by the CHEERS human resources unit?   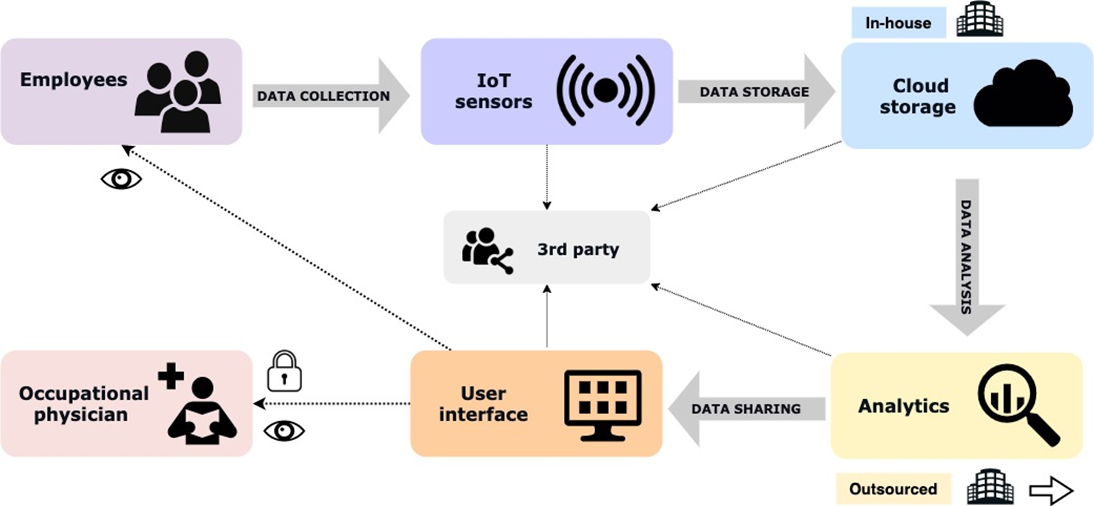 |
| --- |
|  |
| **Scenario II – Step-tracker**  The heads of the company XARIX, after a consultation with two representatives of their employees, have decided to organize “the XARIX steps contest”. The contest is about forming groups of 4 XARIX employees of different age categories. Each group has to collectively make the most steps over a 3 month period. There will be different prizes for groups that manage to increase their mean within-group baseline steps. Steps are monitored with smartwatches produced by the company StepFit. XARIX offers these smartwatches to all employees who are freely motivated to participate in the contest. The aim is to foster teambuilding and motivate employees to engage in more physical activity for health benefits.  The smartwatches can monitor users’ steps, speed of motion, heart rate, body temperature, and blood pressure. Participants’ data are saved on StepFit secured online storage and analysed by StepFit. On a comprehensive app user interface, StepFit outputs personalized reports of users’ step performance, general activity, and global physical health. These reports are privately accessible to users who can, if they wish, share them with their colleagues (by using an « access right option » on the app interface). In addition, StepFit produces a separate anonymized group step-performance report, that is directly sent to the XARIX HR person responsible for the organization of the step contest.  **Discussion Grid**  We will have two kinds of question. I’ll start with quick questions requiring very short one-sentence answers. After that, we will take more time to address more open questions.  **Closed questions:**   - Could this scenario realistically be used in Switzerland? Please respond in one sentence.   - If no, why? (in one to two sentences)   - If yes, do you know Swiss companies using similar IoT technology? Please, provide a yes/no answer and no need to tell names. How many companies? - Could this scenario be legally acceptable in Switzerland? Yes or no? - Would you, as an employee / head of the company XARIX, be in favour of the organization of this step contest? Please respond in one sentence.   **Main open question:**   - In your view, what are the chances (opportunities, advantages) and the ethical issues (possible risks, topic of controversy) related to this scenario?   **Follow up questions:**   - What if reports of step counts become a long-term service at work? - Here is a figure illustrating the data flow in this scenario (see Figure below). Do further issues come to your mind when looking at the data flow process? - What if the occupational physician/HR receive individual reports?   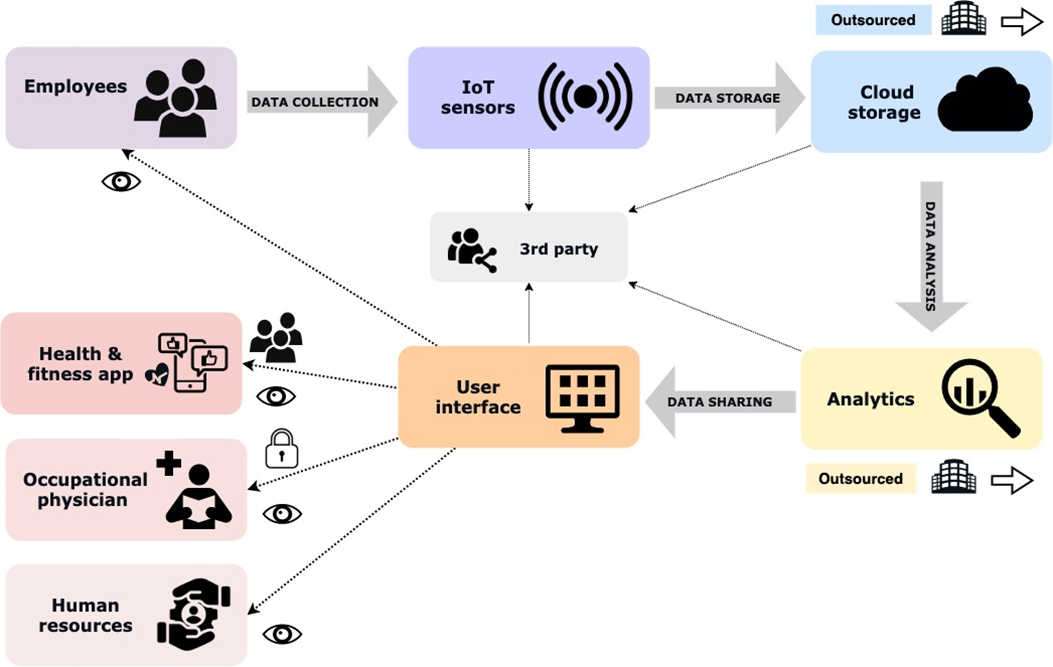 |

| **Scenario III – Sound-tracker**  FINFUTUR is a young startup that has found an interesting but challenging market niche. The perspectives are bright and the heads of the company plan to hire new workforce that need to be flexible and stress resistant. In order to manage the growth of the company while securing attractive working conditions, the heads of FINFUTUR put into place a new working concept allowing employees to freely decide on a day-to-day basis (according to their scheduled appointments and individual organization) whether they will work from home or at work spaces available at the company headquarters.  Since this flexible work solution is quite revolutionary, the heads of the company decide to put into place a monitoring strategy to assess employees’ satisfaction and stress level related to their working conditions and workload. Computers used at the headquarters and at home are equipped with captors of speech tone and speed (disregarding content of speech). The captors are produced by the company FitData. Information collected by the captors is automatically transmitted and stored on FitData’s cloud, then processed by deep learning algorithms, which output an assessment of individual stress level and emotional state. Thanks to an efficient data flow, these algorithms produce real-time signals and recommendations to employees (such as “It may be the right moment for a break”). FitData periodically sends reports of overall stress level to employees. Employees are encouraged to share these reports with their direct supervisors (direct managers, heads of unit) as a basis for discussing their satisfaction with the working conditions and workload. Similar anonymised reports are sent to FINITUR occupational health physician and HR staff.  The HR department oversees presenting the new working concept to FINFUTUR employees. In addition, an anonymous feedback procedure is set up to collect employees’ critical comments.  **Discussion Grid**  We will have two kinds of question. I’ll start with quick questions requiring very short one-sentence answers. After that, we will take more time to address more open questions.  **Closed questions:**   - Could this scenario be realistically used in Switzerland? Please respond in one sentence.   - If no, why? (in one to two sentences)   - If yes, do you know Swiss companies using similar IoT technology? Please, provide a yes/no answer and no need to tell names. How many companies? - Could this scenario be legally acceptable in Switzerland? Yes or no? - Would you, as an employee / head of the company FINFUTUR, be in favour of the use of sound trackers? Please respond in one sentence.   **Main open question:**   - In your view, what are the chances (opportunities, advantages) and the ethical issues (possible risks, topic of controversy) related to this scenario?   **Follow up questions:**   - And what if the computers are also equipped with camera for capturing facial expressions and eye movement? - Here is a figure illustrating the data flow in this scenario (see Figure below). Do further issues come to your mind when looking at the data flow process? - What do you think about the role of the supervisor (direct manager)? - And what if the analytics (data analysis) is done in-house? - What do you think of different consultation procedures (direct consultation of employees themselves, or representatives of employees, or a top-down approach where HR and the CEO make the decision alone)?   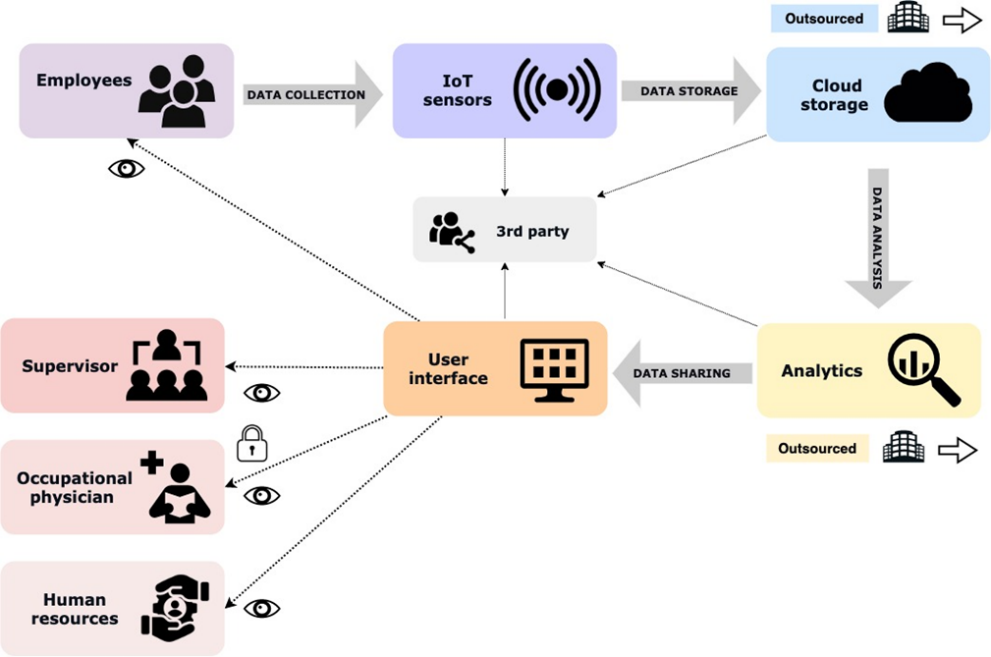 |
| --- |
