# Supplementary material for: The internet of things deployed for occupational health and safety purposes: A qualitative study of opportunities and ethical issues
Source: PLoS One. 2024 Dec 17;19(12):e0315671. doi: 10.1371/journal.pone.0315671 (PMC11651608; doi:10.1371/journal.pone.0315671)
Supplement: S2 Text — (DOCX) [file pone.0315671.s002.docx]

**S2 Text. Full description of qualitative results**

**Contents**

[**1.** **Goal Relevance** 2](#_Toc182404182)

[1.1. Benefits of the device 2](#_Toc182404183)

[1.2. Adequacy of the technology 3](#_Toc182404184)

[1.3. Purposed dual use of the device 4](#_Toc182404185)

[1.4. Limits for the acceptability of IoT for OSH goals 4](#_Toc182404186)

[**2.** **Adverse Side effects** 5](#_Toc182404187)

[2.1. Side effects for employees 5](#_Toc182404188)

[2.2. Side effects for companies 6](#_Toc182404189)

[**3.** **Role of employees** 6](#_Toc182404190)

[3.1. Freedom of choice 6](#_Toc182404191)

[3.2. Information processing 7](#_Toc182404192)

[3.3. Employees’ implication 7](#_Toc182404193)

[**4.** **Data Process** 8](#_Toc182404194)

[4.1. Specific issues related to data flow 8](#_Toc182404195)

[4.2. Data security 9](#_Toc182404196)

[4.3. Lack of trustworthy regulatory actors 9](#_Toc182404197)

[**5.** **Vagueness** 10](#_Toc182404198)

[5.1. Unclear goals 10](#_Toc182404199)

[5.2. Unclear employer’s obligations 10](#_Toc182404200)

[5.3. Uncertainty around the evaluation of the technology 10](#_Toc182404201)

[5.4. Unclear legal issues 11](#_Toc182404202)

[**6.** **Answers to follow-up questions** 11](#_Toc182404203)

[1. Goal relevance 11](#_Toc182404204)

[2. Adverse side effects 12](#_Toc182404205)

[3. Role of employees 12](#_Toc182404206)

[4. Data process 13](#_Toc182404207)

[5. Vagueness 15](#_Toc182404208)

[**7.** **Original quotes in French** 15](#_Toc182404209)

The main manuscript contains simplified maps describing the most relevant codes. Here we present the results with more details. Overall, while evaluating the advantages and ethical issues related to the three scenarios, participants expressed numerous ideas, generating 2202 codes that we grouped into five overarching categories: goal relevance, adverse side effects, role of employees, data process, and vagueness. A further category with 387 codes emerged during the analysis: participants discussed solutions to the ethical issues they raised. Since this topic does not fit with the purported goals of the study, results regarding solutions are kept for a separate publication.

This report of results reflects our two-stage method of analysis: firstly, we present participants’ inputs before we asked follow-up questions (sections 1-5), and secondly, we present participants’ additional inputs resulting from the follow-up discussions (section 6). All French quotations are translated to English, and the original formulas are compiled at the end of this file (section 7).

## **Goal Relevance**

Participants robustly questioned the goals underlying the decision to deploy devices such as those described in the three scenarios (nb-participants=24; nb-occurrences 618). While discussing this issue, they worried about the expected benefits of the devices (nb-participants=24; nb-occurrences=286), their adequacy (nb-participants=23; nb-occurrences=132), and the risk of purposed dual uses (nb-participants=23; nb-occurrences=199). They also expressed various red lines, or limits of acceptability, for OSH goals (nb-participants=11; nb-occurrences=25).

### Benefits of the device

Participants valued the fact that IoTs may provide direct health benefits to employees (nb-participants=21; nb-occurrences=112), either by promoting employees’ global health or well-being, or by addressing specific health issues such as back pain, or stress level. IIp1 for instance note that *“in current societies, we focus too much on the physical side of things. I'm more in favour of developing IoTs or things like that to improve people's psychological health. (...) That's, I think, a priority* [translation from French].” The fact that some devices may provide personalized health support was also valued.

In addition to direct health benefits, participants highlighted that the deployment of IoTs may contribute to health *prevention* and *promotion* (nb-participants=22; nb-occurrences=87), notably by increasing employees’ health literacy, and awareness of the importance of good health, and by empowering them to do so with some sort of assistance. The device could also provide early diagnostics of work-related illnesses. Overall, this could help employees to stay healthy in a preventive way or to avoid risks of accident. In this line, FGp23 said that *“one positive aspect or an opportunity is raising awareness with employees for a healthier lifestyle or how they could include good practices into their everyday life.”* Interestingly, a few participants highlighted that to be effective, heath prevention or promotion should be done under the supervision of a professional counselling, of the sort that occupational physicians could provide. For instance, while discussing the use of the connected chairs (Posture-tracker Scen), IIp2 said: *“So I would say that the chances or the opportunities, (...) it is really the task of the occupational physician or the occupational health team: to try to understand people’s postures, the duration of each posture and then, based on this, to develop a health promotion and prevention campaign. Maybe also to provide guidance, and further training on the gestures and postures to take, and then to go on-site and provide something much more specific and targeted* [translation from French].*”*

Participants also highlighted that, in addition to health, these devices may produce secondary benefits, such as a contribution to employees’ overall quality of life (nb-participants=11; nb-occurrences=20). To illustrate, FGp18 highlights that *“overall as a human being I'll have less back pain and if I have less back pain I'll feel better in my life.”* The devices deployed at work would then have extended benefits in other spheres of life.

At the level of the company, other possible benefits were mentioned (nb-participants=23; nb-occurrences=75), such as a contribution to team building. In this line, while commenting the Step-tracker Scenario, IIp7 said that *“the objective is (...) to encourage team spirit and here is team building. I think it's great (...). So, I think the intention is very good* [translation from French]*.”* Other participants mentioned that the device could help to make managers aware of the importance of employee’s health, and to a series of related benefits such as, increased trust relationships, trust of employees in their company, improvement in working conditions, more equity among employees, reduced costs related to absenteeism, or empowerment and motivation of employees at work. In this line, FGp23 said: *“Their well-being will also translate into more motivation to work as well, as said, as less absent days. More productivity is also good for the company. That is also an opportunity that I see there.”*

Benefits at the societal level were also mentioned, such as a positive downstream effect on public health (nb-participants=6; nb-occurrences=12). As highlighted by IIp13, “*it’s something that’s going to... progressively I think, be part of the digital transformation of our health system... for health purposes* [translation from French]*.”*

Despite acknowledging possible positive effects, we also recorded numerous sceptical thoughts (nb-participants=11; nb-occurrences=26). Some participants expressed doubts that the devices described in our scenario could provide any health benefits, or that, if they did, the effect would be more limited than expected. Some participants, such as IIp2, claimed to *“struggle to view benefits and advantages as sincere* [translation from French]*.”* It was also highlighted that users may fail to comply with the health recommendations of the device (e.g. do more steps, adopt a better sitting posture), or that to express their full potential, the IoTs need to be combined with problematic procedures, such as sharing the reports to employees’ direct managers. Participant IIp12 makes this point: “*the report sent to the employees (...) they're only helpful if they can act on it and then to encourage to share these reports with their supervisors. That is a no! Because it's none of their business.”* Finally, one participant emphasized the difficulty of implementing safeguards around the use of IoTs without impeding the achievement of the intended goals. For instance, if the goal is to track the stress of employees, imposing safeguards against intrusive data capture would make it impossible to achieve this goal.

### Adequacy of the technology

Participants highlighted the importance of evaluating whether the technology is an adequate means to achieve the intended health goal (nb-participants=21; nb-occurrences=69). In our context, such technology should respond to a clear occupational health need. Therefore, employers should first identify workers’ health or security problems and opt for a technology that is necessary and proportionate to the intended goal (nb-participants=14; nb-occurrences=30). Interestingly, half of our participants doubted that the devices discussed in our scenarios are adequate means of tackling the health issues as stake (nb-participants=15; nb-occurrences=39). For instance, IIp4 wondered whether this technology is *“more effective than other measures that could be less invasive.”* Or according to IIp14, *“regardless of the legal aspect, I think that at the moment it is a rather bad idea on several levels to impose the use of connected solutions on employees, especially when (...) we [cannot] argue that there is an absolute necessity to use this kind of system* [translation from French]*.”* Some also highlighted that technological solutions are often too costly to deploy or maintain, or sometimes even outdated for achieving the intended purpose.

In line with this concern, many participants noted the importance of controlling the efficacy and accuracy of the device (nb-participants=21; nb-occurrences=63) with experimental tests and collection of evidence. For instance, while discussing the adequacy of the posture-tracker chairs, IIp13 said that *“if we also have tests, validations, perhaps even clinical ones, which show an advantage over just using these... these chairs, of course I would find it interesting to... to buy chairs like this. Clearly* [translation from French]*.”* However, some expressed concerns about the external validity of those tests: they may show positive results in controlled laboratory contexts, or when the algorithms are fed with standardized data, but fail when applied in the real world. In real companies, employees express a large variety of individual specificities that are hard to capture with simple algorithms. For instance, IIp05 highlights that *“any hint of humour or cynicism are absolutely not... captured by such systems. So in fact, (...) the average individual as he is projected, as he is analysed by these algorithms, is an individual that does not exist* [translation from French]*.”* In a similar vein, several participants worried that the device may not be adequate simply because it is difficult to obtain precise data and to interpret it in a meaningful way to address the targeted health problem. IIp13 explains this difficulty: *“It's gadgetry, it's not precise, it's not medical. (...) Measuring is not difficult. [But] to really measure something that (...) is precise, that makes sense, is much more difficult* [translation from French]*.”*

Interestingly, however, some participants seemed to be less worried about accuracy when they perceived the device as a tool that is already commonly used. This was the case of the Step-tracker Scenario that was deemed more acceptable by several participants, especially by those who reported a personal experience with corporate wellness programs, such as IIp9: *“It's still something didactic, friendly, a non-professional health event that is used in large companies regularly. Every year in many companies. It's called ‘Virgin Contest’ and it works, it works quite well* [translation from French]*.”*

### Purposed dual use of the device

Half of our participants mentioned that despite possible good intentions to promote health at work (nb-participants=15; nb-occurrences=37), employers may in fact be motivated by other purposes for deploying such systems (nb-participants=22; nb-occurrences=164). Some were even more sceptical about the true intentions as, IIp9 who said that *“employers who are sincere and who really want to do something for you, it's (...) quite rare, there's always a hidden objective behind it* [translation from French]*.”*

Participants identified several cases of dual use. Data gathered with these IoTs could be used for organizational purposes such as space management, or for (more or less hidden) managerial purposes, such as surveillance to fight against absenteeism or control workers’ performance and productivity. As highlighted by IIp2: *“the risks are clearly the notion of policing, control, deviation of the tool, (...) for managerial purposes rather than health protection* [translation from French]*.”* In particular, while commenting on the posture-tracker scenario IIp19 asks: *“Is the chair dedicated to one person, so we can know who spends a certain number of hours sitting in the chair? For how long does he get up? Is there a control that is possible by the hierarchical superior who is behind them saying ‘no, but wait, you're supposed to stay seated for 8 hours, but, in reality, you only stayed seated for 6’? By controlling the activity... a little bit of policing, as we call it, which could... which could result from this* [translation from French]*.”*

Participants noted that this risk of surveillance has increased with working from home during the COVID-19 pandemic. Occupational monitoring at a worker’s home with digital technologies has become much more common. As highlighted by IIp9, during the COVID-19 crisis, *“there was no employer who really wanted employees to work from home. Most were reluctant, or at least because you lose some of the control you have over people (...). The manager rarely trusts his employees, and this was a justification for investments, largely devoted to solutions to secure teleworking, so that people can handle sensitive data from home, but also to check whether people who declare having worked 10 hours, or 8 hours, have in reality worked 8* [translation from French]*.”*

Consequently, some participants expressed that the risks of initial dual use purpose, including surveillance purposes, was too high to find such devices acceptable. IIp6 provided a real example of corporate wellness program (similar to our Step-tracker Scen) that was given up because of such dual use risk: *“In one company, they also wanted to use connected watches to measure the number of steps per day, and the watches included a geolocation system... And so, it was obvious that, at any time, with this system, well the employer, if he had access to the data in real time, could know who was where, at what time. And typically, it is easy to track, for example, the number of breaks taken during the day, the duration of the breaks, where people are located, etc. And just, by confronting them [the decision-makers of the company], finally, it [the project] was rapidly abandoned. While the... the initial reason was quite worthy in theory, there were so many risks of deviation that... that it wasn't even worth trying* [translation from French]*.”* On the latter observation, some participants considered that re-purposing the device for surveillance was illegal and dystopic.

However, some participants mentioned that, in specific situations, the dual use of a device might be acceptable. For instance, if the workers’ security is at stake, sound trackers or cameras at work might be justified. Other forms of information tracking may be useful for common good organizational purposes such as for optimizing employees’ working hours or space management. Some participants considered it acceptable to reuse data for the common good, such as for research purposes or for improving the efficiency of algorithms. On the contrary, dual use of employees’ data for the purpose of monetary benefits or targeted advertisement was deemed unacceptable.

### Limits for the acceptability of IoT for OSH goals

Many participants highlighted red lines that should not be crossed, even if one has good OSH reasons to deploy a device (nb-participants=9; nb-occurrences=18). Some participants considered that any objective of re-purposing data primarily aimed at OSH goals is objectionable. Thus, while discussing the reasons for deploying the connected chair (Posture-tracker Scen), IIp9 said that *“the sole purpose of this chair should be the protection of health, and that should be the sole purpose* [translation from French]*.”* Others found that if originally conceived as a short-term intervention (e.g. Step-tracker Scen), such a project is only warranted if it does not turn into a long-term project, because this would generate too many risks (e.g. harming employees, hampering trust relationships). Other participants highlighted the importance of explaining the OSH purpose and implementation procedure to employees to avoid misunderstandings. They also considered that employees should have a right to oppose those objectives and means. Some participants hold the view that all stakeholders should be consulted before the implementation project can be deemed acceptable.

## **Adverse Side effects**

A second major category emerging from our analysis is participants’ concerns about adverse side effects (nb-participants=24; nb-occurrences=251) for the employees (nb-participants=24; nb-occurrences=208), and for the company or society (nb-participants=13; nb-occurrences=47).

### Side effects for employees

Participants articulated the risk that employees could be victims of errors (nb-participants=17; nb-occurrences=48), either directly produced by the system, or resulting from a biased or inaccurate interpretation of the data, leading to erroneous conclusions and possible algorithmic discrimination. FGp21 detailed that *“there are plenty of ways in which the system may not work. Or at least may not work well* [translation from French]*.”* The risk of errors was evaluated as particularly problematic by participants who expressed doubts about the systems’ capacity to account for all relevant individual characteristics of employees (nb-participants=8; nb-occurrences=12). IIp16 explained that *“you can't measure (...) you can't apply something across the board to all individuals!* [translation from French]*.”* Failing to take account of individual characteristics was evaluated as a risk for employees’ individuality.

In the same vein, participants highlighted possible adverse effects occurring in the professional context that could impact employees’ careers (nb-participants=17; nb-occurrences=107). In particular, there is the risk that employers use the data for a secondary purpose once they have it, even if not primarily intended when implementing the system. In that sense, IIp6 mentioned the risk of *“clear diversion of... from the tool's original purpose* [translation from French]*.”*
IIp7 highlighted the lack of control over the final use and reuse of the data: *“On the basis of this [health] principle, everyone is open to receiving all this information. (...) After that, it goes too far! We place sensors in people's homes. (...) They say (...) 'it's just for'[your well-being...]’. But in fact (...), deep down, everything behind it, we don't control it* [translation from French]*.”* In this context, participants enumerated problematic managerial decisions that could be taken based on re-purposed data (nb-participants=9; nb-occurrences=26). Employers could use it for decisions on promotion, termination of contracts or for other forms of managerial control of employees. As highlighted by IIp6, if we consider *“two colleagues under the same conditions, one is more resistant to stress, the other less so, and at some point you have to make a choice about work allocation. If the employer has access to this kind of data, he will logically choose the person who, in theory, is more resistant to stress* [translation from French]*.”*

Work processes and work atmosphere may also be negatively impacted (nb-participants=14; nb-occurrences=26). Employees may be distracted by the device (e.g. by the pop-up health recommendations, or by signals or sounds produced by the system). FGp23 notes that “*as an employee, I feel constantly monitored which would completely occupy my headspace, and I couldn't focus on the actual work I'm supposed to do.”* Employees’ workload or their work performance could be unfavourably reevaluated based on the data collected. As highlighted by IIp10, employers could: *“increase the load on employees if we see that the stress level is low enough in the company. We say, 'Oh well, they're not too stressed, we can increase the level of workload'* [translation from French]*.”* Another concern was that the device might create and increase competition between employees at work (nb-participants=6; nb-occurrences=12). Overall, such adverse effects were suspected to lead to further negative consequences, such as an incentive to cheat with their data in order to increase their performance or to modify their results. Consequently, instead of promoting health or a healthy or team-building environment (Step-tracker Scen), it could hamper relationships between colleagues in the workplace.

Even if deployed for health purposes, the device could lead to adverse side effects on the physical or psychological health of employees caused by the knowledge they are being monitored (nb-participants=15; nb-occurrences=27). As an illustration, FG24 highlights that *“for me, (...) if the machine tells me 'take a break', it's not really going to help me reduce my stress. It's probably going to be even worse* [translation from French]*.”*

Lastly, participants mentioned other possibly unforeseen adverse effects (nb-participants=15; nb-occurrences=36). For instance, the device may further blur the limits between the professional and the private life of employees, especially when it involves extensive surveillance at the workplace of employees’ private spheres and hampers employees’ privacy. As highlighted by IIp14 while commenting on the step-tracker watches: *“Here, the company interferes with whether these employees walk or not... eventually, it's... it goes beyond the professional sphere. And the company starts to monitor in fact the... the behaviour of the employee during but also outside work* [translation from French]*.”* Also, this intense monitoring could shift occupational health responsibility from the employer to the employee, because once the device is deployed, employees seem to be the one in charge of following the health recommendations and maintaining their good health. IIp12 expresses this concern by highlighting that taking care of the health of employees is *“the job of the employer and not the employee, and I do not think that generally it is OK to transmit its obligation (...) to the employee.”* One participant worried about the fact that gamification via IoTs may have the effect of under-evaluating the seriousness of OSH goals in users’ mind.

### Side effects for companies

While discussing difficulties for employers when organizing employees’ work, especially when they are working from home, most participants highlighted the risk that surveillance practices can endanger relationships of trust (nb-participants=12; nb-occurrences=38). The possible dual uses of such devices to the benefit of employers may heighten latent suspicions among employees and lead them to leave the company. Likewise, the companies deploying such devices may suffer from a negative public image, deterring future candidates from applying for a job. According to IIp12 *“I think, (...) you know, the need to survey people at home, how they work, because they can't do it because they're not at work, I think that is really (...) malicious and not good for the work environment and the trust.*”

Further, participants feared that the deployment of such technology at work might increase human-machine dependency which could lead to a dehumanisation of workplace relationships or to problems with the efficiency of OSH measures (nb-participants=4; nb-occurrences=6). This might happen if automated processes replace interactions with OSH representatives or with managers. IIp6 said: *“I find it very (...) automatic and machine-dependent whereas, with stress, [there] is still a human and emotional side, and I'm not sure that managing it in this way is really the... that we succeed in achieving the desired goal* [translation from French]*.”*

Moreover, companies may face unforeseen practical difficulties. For instance, an IoT may be costly to maintain or deploy, and necessitate additional organisational burden, especially when it is deployed in remote contexts (nb-participants=3; nb-occurrences=3).

## **Role of employees**

While reflecting on ethical issues, participants often elaborated on the reactions and roles of employees (nb-participants=24; nb-occurrences=304). From these thoughts, three main subcategories emerged: concerns related to freedom of choice (nb-participants=22; nb-occurrences=80), related to information processing (nb-participants=13; nb-occurrences=26), and related to employees’ implication in the decision process (nb-participants=23; nb-occurrences=211).

### Freedom of choice

Participants stressed the importance of allowing employees the freedom to choose whether to use an IoT (nb participants=21; nb occurrences=56). As mentioned by IIp4, employers should be “*giving people the possibility to choose the kind of measure they want*”, and appropriate measures should be taken to ensure that employees can opt out at any time without any negative impact.

Interestingly, participants also pointed out that, for employees, it may be risky to obtain the right to choose or reject the proposed IoT, whatever decision is taken (nb participants=13; nb occurrences=25). In the case of a wellness program for instance, those who endorse the program may be stigmatized or suffer from discrimination if they do not perform well in comparison to other employees, or if their poor health condition is made more apparent from using the device (nb-participants=8; nb-occurrences=11). For instance, while discussing the autonomy in using the connected chairs (Posture-tracker Scen), IIp7 highlighted that *“the major risk that I see would be, indeed (...) That certain doors would be closed to employees because of bad posture* [translation from French]*.”* And reversely, discrimination or stigmatization may be caused by the decision not to use the device or by a failure to use the device properly (nb-participants=10; nb-occurrences=16). In this context, IIp14 also highlights the case of employees left out even if they are willing to participate to the step contest (Step-tracker Scen): *“[There] are people who are perhaps not very sporty. (...) people with reduced mobility. That excludes them de facto* [translation from French]*.”*

### Information processing

Another topic of concern relates to what should be explained to employees and what is their ability to understand the relevant issues. Some participants explicitly valued the importance of providing comprehensive information and of increasing employees’ ethical awareness (nb participants=7; nb occurrences=9). It makes them more able to assess the benefits and the risks of the technology. This is even more important nowadays, because employees are more aware of data protection issues related to geolocalisation or occupational tracking. As noted by IIp8, *“from an ethical point view, [employees] will [begin to] ask the question ‘but why do you want our data?’ The company[‘s goal is to] track us* [translation from French]*.”* Participants highlighted the related difficulty that employees usually lack digital or technological literacy. Employees may not receive enough clear and understandable information about the device or the data process. Even if informed, they may not be supported in interpreting the data produced by the device, or not trained enough to use the device in a productive manner. For instance, while evaluating the posture-tracker system, IIp14 says: *“Often for all these office chairs they say ‘if used correctly, it can prevent XYZ health problems’. But that's where the problem lies, in using it correctly and without outside help, simply with instructions for use. It's very difficult to know whether or not you are within the.... in the frameworks of this correct use* [translation from French]*.”*

### Employees’ implication

Participants valued the fact of involving employees in the decision process ahead of the deployment of the technology (nb-participants=19; nb-occurrences=72). Some degree of acceptance of the technology was deemed important because employees’ feedback is helpful, and because it is a way to respect them and to ensure their trust at work (even in cases of companies who deploy the technology without consent). Indeed, many participants noted that employees have become sceptical in view of the regular scandals in the news related to private data leaks or abusive occupational surveillance; this is why it is important to involve them in the decision procedure (nb-participants=12; nb-occurrences=28). IIp1 expresses this distrust as follow: *“If we tell people ‘yes, but don't worry, the data is anonymised (...)’ I don't think people believe it anymore. Honestly speaking, it's a lot of hot air. Even if it's true, if the company is doing everything right, there's a doubt* [translation from French]*.”*

However, participants also highlighted that employers tend to avoid consultations because they fear employees’ refusal (nb-participants=16; nb-occurrences=25). Moreover, even when information and consultation processes are properly conducted, they might be biased by dependency relationships: employees may not be free during the discussion process because of their subordinate status, or because the trust relationship depends on interpersonal factors (e.g. colleagues’ attitude). The consultation process may also favour employees that are already healthy or taking care of their health. For instance, participation and implication of employees already in good health may be higher in wellness step-count programs, compared to the employees who would benefit the most from the device. Thus, the engagement in the decision procedure (and thus power in the end-decision) may not be equally distributed.

In addition to consultation procedures, participants discussed the issue of employee consent (nb-participants=23; nb-occurrences=142). While they unanimously appreciated the idea of seeking consent from employees, especially when medical data are at stake, they also highlighted reasons to doubt the value of that consent.

Firstly, employees’ evaluation of the technology could be impaired because they are already accustomed to the proliferation of technological and monitoring techniques; this makes it difficult for them to properly assess the risks of such technology (nb-participants=19; nb-occurrences=72). Indeed, some monitoring techniques are already commonly deployed at work or in private contexts, such as step-tracker watches and wellness programs. IIp1 expresses this point as follow: *“people in societies, I would say Swiss, French, (...) are quite accustomed now to everything that is technology (...) So they won't be so afraid of (...) this kind of thing* [translation from French]*.”* In this context, it was highlighted that the Covid-19 pandemic and the deployment of occupational monitoring devices at home, or the trivialization of personal connected devices such as home assistant or smart-watches contributes to the development of employees’ acceptance of digital tracking (nb-participants=12; nb-occurrences=14).

Second, employees’ lack of freedom in the workplace decreases the relevance of their consent (nb-participants=18; nb-occurrences=49). Employees may feel pressured to accept the deployment of an IoT, or their consent may result from unbalanced incentives. As clearly expressed by IIp12, “*we as employees, we depend on the employers, so he's the one paying our salaries. And if he's introducing a new system like a new computer or whatever, and we refuse to use that system, we usually lose jobs.”*

Lastly, participants highlighted that consent may be wrongly considered the key solution to ethical issues, casting a shadow over other important difficulties (nb-participants=10; nb-occurrences=24). For instance, once they have consented, employees may face difficulties and challenges in exercising their rights of access or control of the processing of their data, particularity problematic when such data is shared with third-parties or when the question of data ownership remains unclear. It may also be difficult for employees to ask employers to provide transparent information on which to base their consent. To illustrate, IIp17 said: *“I would not dare to ask my employer because I wouldn’t want him to take it the wrong way. I would trust him, obviously, not to do anything wrong with my computer, but still, the problem remains* [translation from French]*.”* Further, it may be practically difficult to gather proper consent from all relevant persons while deploying an IoT. For instance, when a device is deployed at employees’ home, other family members, and guests should be asked for consent, but this is practically impossible. Similar concerns apply when the device is deployed in an open space where many passing people might be recorded by the device without having the opportunity to consent. This concern is expressed by IIp4: *“I mean, I would have probably to inform every single person I interact with, yeah, according whether they agree with that. I mean, it's just not doable…”*

## **Data Process**

Another important set of concerns discussed by participants is related to the data process (nb-participants=23; nb-occurrences=274). Three subcategories emerged: specific issues related to the various stages of the data flow, including data collection, sharing, storage, analysis, and governance (nb-participants=22; nb-occurrences=207), the general issue of data security (nb-participants=16; nb-occurrences=54), and the lack of a trustworthy actors (nb-participants=8; nb-occurrences=20).

### Specific issues related to data flow

Data Collection (nb-participants=19; nb-occurrences=82). According to some participants, data collection is more acceptable in the private than the professional sphere because in private contexts individuals are solely responsible for their choices to buy and use an IoT. Moreover, when these devices are deployed in the workplace, participants emphasized the importance of data minimization (i.e. collection of personal information should be limited to what is directly relevant and necessary to accomplish the specified purpose), and the difficulty of evaluating whether this principle is respected in concrete cases, as in our three scenarios. The risk of infringing the principle of data minimization with massive and wide spectrum data mining was evaluated as high, and particularly problematic when done in home office contexts. As expressed by FGp18 while discussing the sound-tracking scenario, *“this continuity of sound capture in another private home also raises a number of questions”.* Moreover, participants highlighted that risks are particularly high when the devices collect sensitive health and biometric data, as well as behavioural data, that can be crossed at the analytic stage.

Data Sharing (nb-participants=17; nb-occurrences=85). The most discussed concern related to who the data are actively shared with or transferred to (nb-participants=17; nb-occurrences=78). Overall, because of its sensitive nature, participants considered that it should not be sent to employers or to third parties external to the company (except for the dedicated purpose of analysing them). Further, several participants made it explicit that HR should not access the data, especially when it is not of obvious use for OSH purposes. They justified this by claiming that the HR department is more likely to act in the interest of the employer, and less for the employees, as expressed by IIp6, an occupational nurse: *“In my practice, I have been confronted on a number of occasions with a clearly inadequate interest on the part of (...) Human Resources or management, (...) the access, well the reason for the access was clearly not (...) prevention, but it was really for... control of the control of employees* [translation from French]*.”* Participants also discussed possible exceptions. Some considered it acceptable (or even recommended) to share *personal* data with employees and with the occupational physician because it would help him or her to provide health recommendations or feedback. Moreover, sharing *aggregated* data with the HR department was deemed more acceptable, even if risky. Some participants also expressed fear about data being shared with others internal to the company, such as colleagues or an employee’s direct manager. Overall, however, they elaborated little on the reasons for restricting data sharing to third-parties.

Data Storage (nb-participants=15; nb-occurrences=30). Some participants were concerned about the storage location and expressed the need to introduce safeguards. A preference for a storage in Switzerland rather than in a foreign country was expressed, especially when dealing with sensitive health data.

Data Analysis (nb-participants=7; nb-occurrences=10). A few participants expressed concerns about the possible re-purposing of the data by external companies whose task is to analyse the data. In this context, IIp5 underlined that in the Step-tracker Scenario, *“it seems fishy to me that this company* [the one responsible for the data analysis] *doesn't do anything with the data* [translation from French]*.”* In this context, as expressed by IIp5, participants found it important *“to have a little more transparency, a little more information on how the analysis is done* [translation from French]*.”* Three further participants highlighted that HR should not oversee the analysis due to the dual use risks, and another participant preferred external analysis to internal.

Data governance (nb-participants=7; nb-occurrences=11). Participants emphasized that is it unclear who oversees the overall management of the data flow, and if such a governance is feasible. It was also highlighted that lack of governance makes it difficult to evaluate who is entitled to access the data, or how to set up internal processes providing authorization to access the data.

### Data security

Participants identified data security as a major concern, especially given the sensitive nature of the medical data collected. They discussed the risks of a cyberattack, data leak, data loss, or lack of data confidentiality (nb-participants=8; nb-occurrences=10). Participants also feared that full data anonymisation was not possible, especially when IoTs are deployed in small companies composed of a limited number of workers (nb-participants=9; nb-occurrences=16). IIp14 highlights that *“at the end we have the report which is sent to Human Resources, so... and then, even if it's anonymized, I think that Human Resources could more or less know who has chosen to be part of this program or not”*. In such situations, HR could match information already possessed on employees with the data collected for OSH purposes. The process under which data were *de facto* accessible (even if not explicitly shared with) was also a source of concern (nb-participants=5; nb-occurrences=12). To illustrate, while discussing the posture-tracker scenario, IIp6 worried about *“who has access to the data? BioDat? FitChairs? The occupational physician, the company, Human Resources? It's not, it's not clear* [translation from French]*.”*

### Lack of trustworthy regulatory actors

Participants identified several trustworthy actors in Switzerland who can regulate the data process: the Federal Office of Public Health, cantonal authorities, the foundation *Promotion Santé Suisse* (mandated by the cantonal authorities and health insurance companies to stimulate, coordinate and evaluate measures aimed at promoting health in Switzerland) and occupational physicians. However, they worried about the extent to which they are sufficient gatekeepers in particular cases such as those described in our scenarios (nb-participants=4; nb-occurrences=7). For instance, it was noted that occupational physicians and nurses have limited power or are consulted too late, once the technology is already deployed, making it difficult for them to play a decisive role. IIp6 makes it clear*: “We are never a mandatory safeguard because, when there are projects, we are never considered to be someone who should give her opinion regardless of the type of project, no. Sometimes we are informed beforehand because people realize that it is a project that affects health or involves medical data, and* (...)*, sometimes not at all, and then we learn a few weeks or a few months after the implementation of the project that it exists* [translation from French]*.”*

## **Vagueness**

While discussing ethical issues, participants expressed numerous uncertainties. This pattern was recurrent in all interviews (nb-participants=24; nb-occurrences=169) and became an independent *vagueness* category in our analysis. Even if vagueness is not in itself, an ethical issue, it forms the basis of ethical questioning or issues highlighted by participants.

Issues about vagueness were discussed under the four other overarching categories of codes described above. To recap, here is a summary of the main points that have been raised. While discussing *goal relevance* issues, participants worried that IoTs are conducive to wrong dual use purposes that are difficult to detect and assess, especially ahead of the deployment of the technology. They also noted how difficult it is to evaluate who (employees, the company) benefits or should benefit from the technology, how and to what extent. While discussing *adverse side effects*, participants wondered about the long-term effects of the use of IoTs on workers. Will the health benefits be counterbalanced by other adverse effects on their health? Moreover, participants wondered how such technology, once deployed, might impact the company overall and society as a whole. While discussing the *role of employees*, participants noticed how difficult it was to evaluate whether a meaningful consent can be obtained in a workplace context. How it is possible, in practice, to guarantee the rights of people impacted by the IoT (employees, their family, or any subject to the device), to properly inform them, obtain a valuable consent, or grant them access and control over their data? While discussing *data process* issues, participants raised numerous questions such as: Who is in charge of data analysis? What is the extent and type of data collection? How is data collected? Who has or should have control over the data? What data are shared with who and where is it stored? Is there anyone trustworthy capable of playing a sufficient gate-keeping role? They were insecure about the involvement of different actors during the data process, and about whether this could be made transparent.

In addition, further ethically relevant domains of vagueness emerged from participants’ inputs: the goals underlying the deployment of the technology were unspecified (nb-participants=20; nb-occurrences=39), obligations for employers were unclear (nb-participants=14; nb-occurrences=37), it was difficult to evaluate the product’s adequacy at different stages of its life-cycle, from its development to its post-implementation evaluation (nb-participants=18; nb-occurrences=57), and overall, many uncertainties remain related to legal issues (nb-participants=11; nb-occurrences=39).

### Unclear goals

While discussing our scenarios, participants worried that in practice, the motivation of decision-makers underlying the deployment of such devices may be underspecified. When deciding to deploy a device, employers may have unclear intentions (e.g. unclearly defined OSH goals), or base their decision on unclear evidence that the device is effective.

### Unclear employer’s obligations

Participants were unsure about how much employers are expected to protect employees and to promote their health. For instance, some participants wondered or doubted whether employers should intervene with the specific health problems that were described in our scenarios (nb-participants=5; nb-occurrences=16). IIp12 expresses it as follows: *“from a public health perspective, I think it's good to make people move more and it's good to make them aware of what they eat and all those things. I completely agree, but I do not think it's a company task to do that”*. Similarly, IIp4 expresses how difficult it is to set clear obligations for the employers: “*And then I mean the general risk [is] finding this boundary between what's the company supposed to [do and...] how far is the company supposed to (...) go for supporting employees”*.

### Uncertainty around the evaluation of the technology

Participants considered that in many cases, the relevance (best means to achieve the intended benefits), effectiveness and reliability of the technology was unclear. For instance, they worried about the risk of inaccurate outputs of newly developed devices, especially if they include algorithms trained on irrelevant datasets. Participants also expressed concerns about the possibility of determining relevant evaluation criteria to properly assess the device. They worried about the lack of clinical or scientific tests, making it difficult to assess the appropriateness of deploying such devices. Once deployed, it remains difficult to evaluate whether the devices fulfil the aimed OSH purposes (nb-participants=16; nb-occurrences=36).

In this context, they pointed out that companies are often pressured by market competition, and over-optimistic about the success of their technology (nb-participants=10; nb-occurrences=17). This leads them to develop and deploy new devices at a fast pace, without much critical thinking, evaluation, and safeguarding measures, or to hastily assume that a technology already deployed elsewhere can be safely and legally implemented. Other participants pointed out the risk of techno-solutionism, overoptimism about the usefulness of the technology. This is nicely expressed by IIp6: *“Knowing that obviously, I'm not at all opposed to high-tech and the development of these technologies, but sometimes we first develop the technology and the data collection, then we ask ourselves the question of whether it's relevant or not? Then, as we have developed the thing, well, at the marketing level, we have to sell the product, and then we try to cobble together a use for what we have developed and we ‘pretend that’. And in this scenario, I have the impression that we're rather on that side of things* [translation from French]*.”*

### Unclear legal issues

In the same vein, some participants found that current laws regulating the deployment of IoTs are unclear and difficult to interpret (nb-participants=7; nb-occurrences=25). Several participants emphasized the importance of the General Data Protection Regulation (GDPR) and the Swiss data protection law (LPD). Simultaneously, they complained that Swiss law is insufficient, unclear, weak, permissive, and lacks effective safeguards, in comparison to European law. However, they could not provide clear examples to illustrate such critical points (note that our interviews took place before the enforcement of a new version of the LPD). IIp9 underlined that: *“The problem is that in Switzerland we are in a legal no-man's land on employees’ surveillance. (...) There is little case law and there are few resources* [translation from French]*.”* Some participants said that in the Swiss legal context, it is unclear which technological deployment would fulfil OSH objectives and would be acceptable from a legal point of view. It was also emphasized that the issues at stake are particularly difficult to deal with, because data often cross-national boundaries.

Participants worried also about the extent to which the data protection law is respected. They highlighted the importance of respecting data protection and providing strong guarantees while implementing IoTs. But they suspected that legal issues are often not considered during the design of the data process. Moreover, uncertainty was expressed about how employees can have ownership over their data at any stage of the process. Participants also wondered if and how long it is legal to store the collected data. While discussing these issues, they expressed uncertainties, such as whether data analysis made by a non-Swiss external company is legal (or not), or whether occupational physicians are obliged to receive training on the data protection law.

## **Answers to follow-up questions**

While answering more specific follow-up questions or looking at the figures illustrating the data flow related to each scenario, participants provided additional input. Overall, the follow-up process increased the total number of codes up to 2202. Among these codes, some items were reiteration of existing codes, and 883 items were new ground-level codes not mentioned spontaneously during participants’ preliminary assessment of the ethical issues. These new codes fit coherently within the five major categories described above (goal relevance=205; adverse side effects=133; role of employees=99; vagueness=41; data process=510) and bring additional theoretical material discussed below. Moreover, in some cases, existing codes that were only mentioned by one or few participants in the main dataset became significantly more “grounded” (i.e. mentioned by a much higher number of participants) during the follow-up process. In what follows, we present the most noteworthy cases.

### Goal relevance

Follow-up questions generated additional thoughts on the positive effects of IoTs for public health and for society (before: nb-participants=6 & nb-occurrences=12; after follow-up: nb-participants=11 & nb-occurrences=21). Notably, participants highlighted that aggregated data of large groups can be used to design and evaluate health prevention or ecological measures. For instance, when asked in the sound-tracker scenario to assess a more invasive technology (a facial expression tracker), some participants highlighted that the collection of more complete and accurate data increases the quality of the analysis, which in turn can help to prevent health issues and optimize healthy working conditions for employees.

Except for these positive considerations, participants mostly discussed ethical difficulties. Notably, they were much concerned with the adequacy of the technology for OSH purposes (before: nb-participants=23 & nb-occurrences=132; after follow-up: nb-participants=24 & nb-occurrences=223). New reasons were provided: for instance, some IoTs may be useful for one type of employee but inapplicable to other types (e.g. those who do not work in an office), creating inequitable OSH programs. Some participants found that replacing management based on trust relationships with technology is an inadequate aim. Another participant highlighted the pervasive aspect of the use of IoTs, because they seem attractive and harmless at first glance, and this appearance conceals many problematic aspects that have already been discussed.

Interestingly, the follow-up question made it more explicit that some participants fundamentally diverged in their evaluation of invasive IoTs. In response to the notion of deploying facial recognition technology (Sound-tracker Scen), 3 participants said they would not oppose it if these devices truly served OSH purposes, or if they provided efficient responses to other managerial concerns, such as decreased productivity when working from home. Conversely, 14 participants explicitly evaluated such technology as inadequate, arguing that it was illegal, and would increase risks of surveillance and problematic dual data use. When discussing this example of invasive technology, participants became more explicit about why the necessity principle may not be met with IoTs, and about the sort of harm that may occur. For instance, FGp23 explains that “*if you start collecting the visual input as well it is an even bigger intrusion (...) than just being able to listen. Because I could be next door and hear what you are saying (...), but you could be naked and I don't know. But if you're doing this with the camera on it’s quite a different thing.”* Similarly, in response to the idea of extending the use of an IoT over the time initially planned (Step-tracker Scen), 2 participants did not object to the idea, while the others stressed more vividly that IoTs deployed with short-term aims should not become long-term.

### Adverse side effects

The follow-up question about the deployment of a more invasive IoT (facial expression tracker) helped participants to develop more precise thoughts on the adverse effects of IoTs (before: nb-participants=24 & nb-occurrences=251; after follow-up: nb-participants=24 & nb-occurrences=384). If deployed long-term, IoTs could have unwanted influence on employees’ behaviour, leading them to develop more refined cheating habits or constraining their natural spontaneity. Extending the latter line of thought, IIp20 notes that *“the main side-effect of Big Data that we haven't really felt yet (...) is the erasure of singularity. And that's something that's going to happen, and it's hard to say what the consequences will be* [translation from French]*.”* An increased use of IoTs could make surveillance more common at the societal and work-place level, and lead to a dangerous pressure to conform, e.g. to adopt a ‘healthy’ behaviour, without any guarantee that the IoT will improve health. Finally, participants discussed the risk that intrusive surveillance devices could endanger employees’ autonomous working habits (e.g. by reducing the flexibility of their working hours). At the societal level, the use of these devices could create new environmental and ecological issues (e.g. electricity costs).

### Role of employees

In response to a follow-up question sounding out what participants thought of consultation procedures ahead of the deployment of a device, they came up with more to say on the topic (before: nb-participants=16 & nb-occurrences=25; after follow-up: nb-participants=20 & nb-occurrences=65). They added reasons for the importance of consultation processes: sensitive medical data are at stake; the processes increase transparency and employees’ adherence to the project, which is of practical use. If a device is imposed without consultation, it could hamper existing trust relationships between the company and its employees.

In the follow-up process, more participants insisted on the importance of transparency, comprehensiveness of information, and the formal commitment of employers. However, many remained strikingly vague about whether (and about how many) employees should have a say in the decision to deploy the technology. Here is an example by IIp10 *“If it's discussed with the employees and there's, I don't know, a charter, something that... that gives guarantees that... well, once again, you can (...) never rule out abuses! But (...) it's this benefit-risk ratio: if the guarantees are sufficient, we can say ‘yes, well, there you go’* [translation from French]*.”* Some participants doubted the relevance of in-depth democratic consultation. They highlighted that trust and acceptability can be ensured if employee representatives or others who can advocate the employees’ point of view are consulted before the deployment of the project, or by safeguards and guarantees. Other participants pointed out the limitations of consultation and information. Even if most employees are correctly informed and agree with the deployment of an IoT, there will always be some who disagree, which is problematic. As explained by IIp16, if you are in such a situation *“It changes a lot because you lose your free will. You can no longer say 'no, I object!* [translation from French]*.”* They pointed out that transparency and the formal engagement of employers may not be enough to guarantee trust. Fatalistic thoughts emerged: the devices will be imposed on employees regardless without consultation.

Contextual sensitivity was highlighted: once aware of OSH goals and how the technology is deployed, employees can be reluctant to share their personal data in the work context, even if they are without qualms about sharing the same data in private contexts. As explained by IIp13: *“I have the impression that today, they don't realize (...) that on their own, they're already sharing a huge amount of private personal data! (...) And then, on the other hand, as soon as a specific tool is implemented, they immediately raise the issue of all the sensitivity of their data. This is just contradictory on the principle. That's the big problem today* [translation from French]*.”*

### Data process

The figures of the data flow that we showed in relation to each scenario and some follow-up questions (e.g. would a facial recognition tracker be acceptable? What if data is shared with the occupational physician, HR, or a direct supervisor?) led to a large increase in participants’ inputs (before: nb-participants=23 & nb-occurrences=274; after follow-up: nb-participants=24 & nb-occurrences=784).

Although few participants initially expressed worries related to the transmission of data to external partners for analysis, the follow-up inputs provided made them more aware that it counted as *data sharing* with third parties. Consequently, they became more aware of correlated risks of re-purposing (before: nb-participants=8 & nb-occurrences=9; after follow-up: nb-participants=16 & nb-occurrences=51). In this context, more participants found it unacceptable for a third-party analysis company to sell the data to further third parties (nb-participants=13; nb-occurrences=38). Some participants considered data transfer acceptable if data was anonymized and if the aim was improvement of the algorithms or overall quality of the technology (nb-participants=6; nb-occurrences=8).

Participants elaborated further on why it could be appropriate to *share data with the occupational physicians* or nurses (nb-participants=14; nb-occurrences=49): their professional knowledge make them capable of detecting incorrect or poor-quality data, of interpreting the data, and of using it in a meaningful way for OSH purposes. With this information, they can detect employees’ frailties or illnesses and provide individualized help; they can detect group-level patterns to be addressed with global prevention measures. They can also help employees to interpret individual reports generated by IoTs. Despite these reasons for trusting occupational physicians, some participants expressed discomfort at the idea of sharing such sensitive data with them (nb-participants=6; nb-occurrences=8).

Participants explained in more detail why, despite acknowledging the opportunities for health prevention, they worried about the idea of sharing employees’ data with internal actors such as employees’ direct managers, or HR. In relation to *direct managers* (nb-participants=17; nb-occurrences=77), participants noted that they may play a positive role in identifying security and health risks factors, in relaying the needs of employees at higher hierarchical levels, and in contributing to the design of health strategies adapted to the actual work context. Despite this key role of intermediary between employees and the head of the company, they were not considered the right people to receive sensitive data and reports (nb-participants=15; nb-occurrences=47); they often do not have enough power within the company to create organisational changes in favour of employees. As illustrated by IIp 11: *“Then his collaborator comes to him and says, 'I am not satisfied with the conditions,' which will only generate frustration because the supervisor can only respond by saying, 'Well, for now, we cannot change [these conditions... and] the workload remains the same. Ok!’”* It was highlighted that direct managers work closely with employees in their teams, making it difficult for subordinates to refuse demands to use an IoT and to share the data collected. Moreover, data sharing could be experienced as very intrusive by employees, increase their level of stress, and hamper the working relationship. While discussing concerns related to HR (nb-participants=20; nb-occurrences=83), participants pointed out that they may not have the knowledge and capacities to accurately interpret the data. This would increase the occurrence or gravity of wrong dual use, re-purposing practices, or undue sharing of sensitive data.

The issues related to *data storage* received more attention during the follow-up process (before: nb-participants=14 & nb-occurrences=22; after follow-up: nb-participants=19 & nb-occurrences=48). Beyond merely expressing preferences about where data should be located, some participants expressed a view on who should oversee storing it. Interestingly, these participants stated that external bodies (including GAFAM) are better equipped to securely store data than the companies themselves who are vulnerable to hackers; others said that the issue of where to store the data (in the company *versus* externally) was of secondary or no importance.

Only when explicitly asked about who should perform *data analysis* (should this task be outsourced to a third-party or done in-house?), did participants begin to express related concerns (before: nb-participants=7 & nb-occurrences=10; after follow-up: nb-participants=21 & nb-occurrences=115). Most participants found it highly problematic for data analysis to be done in-house and grounded this evaluation on their suspicions against HR; they worried about their competence to perform analysis and about the risk of re-purposing and dual use of the data. External companies were seen as more competent, neutral and likely to ensure confidentiality and data safety. Given a lack of internal competencies, companies may have no choice but to outsource data analysis. Only a few participants expressed concerns about externalization, notably the risk of data reselling and breach of confidential information (e.g. when non-aggregated and personal data are transferred). Some participants were slightly more open to the idea of in-house analysis but on strict condition that the data were used for health purposes only. Four participants made no comment about this aspect of the data process. Finally, it was considered better to analyse aggregated data. Some participants expressed general concerns about the likelihood of accurate interpretation of the data, especially when characteristics open to interpretation (such as stress level) are measured. Participants expressed concern about the issue of *data robustness and interpretability* (nb-participants=4; nb-occurrences=5)*.* The data collected may not be sufficiently precise, reliable, or even interpretable. They also worried about the quality of the subsequent analysis based on poor data: it may fail to consider important contextual or individual characteristics of employees or lack diversity.

New issues related to *overall data governance* were raised (nb-participants=14; nb-occurrences=35). Participants expressed concerns about who should exert control over the data flow, or governance over the exploitation of the data. It was highlighted that too many intermediaries are involved at different stages of the process, and that lack of transparency could be a major issue. Specifically, they highlighted the difficulty of interpreting the data in a meaningful way; when asked to evaluate with whom the data could be shared, some participants were unsure due to their incomplete understanding and preferred to withhold ethical evaluation.

During follow-up discussions, several participants elaborated more on the idea of *trustworthy actors* (before: nb-participants=8 & nb-occurrences=20; after follow-up: nb-participants=17 & nb-occurrences=52). Despite the above-mentioned risks of abusive data sharing, overall, participants highlighted the trustworthiness of *occupational physicians*, as long as they remained independent from the influence of the employer or HR. They are bound to medical secrecy and their professional role makes them more likely to use the data in a more responsible manner for OSH purposes. Other participants highlighted the adverse effect of some organizational and managerial contexts: for instance, if occupational physicians have strong relational ties with managers and HR, it increases the risk that employees’ data could end up in the wrong hands and be used for the wrong purposes. IIp17 expresses this as follows: *“I don't know [anything] at all, (...) but I think that he...she risks being a kind of puppet finally in the human resources department which puts so much pressure because the management bothers so much about results, concrete effects and measures which are taken.”* Participants discussed the dilemma of occupational physicians needing to know more to treat better while being possibly influenced by powerful actors in the company.

Participants also elaborated on the lack of *competent authorities* for the promotion and regulation of OSH. Some participants said that there was not enough political will to enforce existing public institutions, such as the national data protection authority, and a lack of nationally organized solutions or local webs of trustworthy actors in the field of OSH. Even existing actors are difficult to identify and to contact when needed. Therefore, small companies have limited possibilities of offering high quality OSH services. Only big private companies that follow international standards end up taking effective OSH measures, and in that case, given the lack of control by external trustworthy actors, it may not always be clear whether these initiatives can be trusted. This last worry is expressed by FGp22: *“If* Promotion Santé *[a health promotion organisation] proposed such a kind of offer [deployment of an IoT for OSH purpose] for large companies, (...) would have a lot more trust because it also has the duty to ensure that (...) such a project follows the purposes of the organisation [*Promotion Santé*]*” [translation from French].

### Vagueness

Issues of vagueness were discussed in the follow-up within the other overarching categories. While discussing goal relevance, participants highlighted the uncertainties surrounding the benefits of the device, the intended purposes, the possible dual use and the adequacy of the technology. During discussion of the adverse effects, participants highlighted that it was hard to predict the long-term effects of the technology. When considering the role of employees, many participants were unsure about whether and how employees should be consulted. In the discussion of the data process, it was highlighted that certain actors (notably HR) may not have the relevant knowledge to make adequate decisions or may have difficult conflict of interests (notably, occupational physicians). During the follow-up process, we observed an important *increase of number codes* related to lack of clarity of the data process governance (before: nb-participants=7 & nb-occurrences=11; after follow-up: nb-participants=14 & nb-occurrences=35). Discomfort was expressed more vividly about understanding how data are stored, managed, analysed, interpreted and transmitted, increasingly difficulty in identifying ethical problems.

Furthermore, participants were more explicit about the difficulty in identifying clear obligations for the different stakeholders involved (before: nb-participants=14 & nb-occurrences=37; after follow-up: nb-participants=16 & nb-occurrences=48). This led them to raise numerous questions and discuss possibilities about which they had no firm opinion. To what extent are employers responsible for employees’ health? Does it extend to the promotion of health self-management? Another question was related to political responsibility: one participant complained of a reluctance to regulate technologies at the political level, which increases the difficulty of evaluating who is responsible in the case of adverse effects due to the use of IoTs. More generally, in a context of proliferation of these technologies in an unregulated context, employers may be in doubt about whether the deployment of connected devices for OSH purposes is acceptable and fulfils the legal requirements. If an audit procedure is currently conducted in a company by a public administration, it is unclear which evaluation criteria will be used to control and assess the acceptability of IoTs used for OSH purposes.

Moreover, some participants (nb=2) wondered whether stronger regulation, similar to the new European regulation for medical devices, would be helpful. A worry was that it would add further complexity in the evaluation of stakeholders’ duties and responsibilities, and thereby hinder the development and deployment of new technologies.

## **Original quotes in French**

**Section “Goal relevance / Benefits of the device”**

IIp1: Il développe que *“on se focalise, beaucoup trop, dans les sociétés actuelles, sur le côté physique, des choses. Moi, je suis plus d’avis de développer de l’IoT ou des choses comme ça pour améliorer la santé psychique des gens  (...) Ça c'est, je pense, c'est une priorité.”*

IIp2: Elle souligne les opportunités suivantes: “*Alors je dirais que les chances ou les opportunités, j'ai envie de dire positives qui pourraient partir, c'est vraiment pour le médecin du travail ou l'équipe de la santé au travail, d'essayer de comprendre les positions que prennent les gens, la durée de chaque position et puis, derrière, développer une campagne de promotion de la santé et de prévention, peut être aussi d'orienter, et après une formation sur les gestes et postures, et puis d'aller sur place avec quelque chose de beaucoup plus spécifique et de ciblé.”*

Ip7: Elle souligne que *“l’objectif est (...) de favoriser l'esprit d'équipe et puis voilà un team building, je trouve ça super (...) donc je trouve l’intention derrière très bonne.”*

IIp13: Il explique: *“Et puis c'est quelque chose qui va… Qui va gentiment, je pense, ça fait partie de la transformation digitale de notre de notre système santé… sanitaire.”*

IIp2: Elle a *“de la peine à avoir les bénéfices et les avantages pour être sincère.”*

**Section “Goal relevance / Adequacy of the technology”**

IIp14: Il explique: *“indépendamment du volet légal, je pense que actuellement c'est une assez mauvaise idée à plusieurs niveaux, d'imposer l'utilisation de solutions connectées aux employés, surtout quand [...] on peut pas argumenter qu'il y ait une nécessité absolue de d'avoir recours à ce genre de système.”*

IIp13: Il souligne que*“si… on a aussi des tests, des validations aussi peut être aussi cliniques, hein, qui montrent un avantage par rapport juste pour l'utilisation de ces…de ces chaises, bien sûr que je trouverais intéressant de de… d'acheter des chaises comme ça. C'est clair.”*

IIp5: Il explique: *“... un peu d'humour et du cynisme sont absolument pas… saisies par ce type-là. Donc en fait, c'est l'individu tel qu'il est projeté, l'individu moyen tel qu'il est projeté, tel qu'il est analysé par ces algorithmes-là, est un individu qui n'existe pas.”*

IIp13: Il explique que *“c'est du gadget, c'est pas précis, c'est pas médical. (...) Mesurer c'est pas difficile. Mesurer vraiment quelque chose qui (...) est précis, qui fait du sens, c'est beaucoup plus difficile.”*

IIp9: Il explique: *“Ça reste quelque chose de de didactique, de sympathique, un événement santé non-professionnel qui est utilisé dans les grandes entreprises régulièrement. Chaque année dans plein d'entreprises. Ça s'appelle Virgin Contest et ça marche, ça marche plutôt bien.”*

**Section “Goal relevance / Purposed Dual Use of the Device”**

IIp9: Il alerte sur le fait que *“les employeurs qui sont sincères et qui veulent réellement faire quelque chose pour vous, (...) c'est (...) assez rare, il y a toujours un objectif caché derrière.”*

IIp2: Elle souligne que *“les risques c'est clairement la notion de flicage, de contrôle, de déviation de l'outil, (...) à des fins managériales plus que protection de la santé.”*

FGp19: Il souligne qu’*“il y a toute la question aussi du : est-ce que la chaise est dédiée à une personne et du coup on peut savoir qui passe combien d'heures assis sur sa chaise ? Combien de temps est-ce qu'il se lève ? Est-ce que y a du coup un contrôle qui est possible par le supérieur hiérarchique qui est derrière va dire ‘non mais attends, t'es censé rester assis 8h mais concrètement t'es resté assis que 6’ enfin voilà. Un contrôle de l'activité…  un peu le flicage comme on l'appelle, qui pourrait… qui pourrait en découler. ”*

IIp9: Il souligne qu’*“ il y a aucun employeur réellement qui a souhaité réellement que les collaborateurs partent en télétravail. La plupart étaient réticents, ou en tout cas parce qu’on perd une certaine partie du contrôle qu'on a sur les gens (...). Le manager a rarement une grande confiance en ses collaborateurs et ça a justifié l'investissement en grande partie de solutions à la fois pour sécuriser le télétravail, pour que les gens puissent manipuler des données sensibles depuis leur domicile, mais aussi pour surveiller que les gens qui nous déclarent qu'ils ont fait 10h de travail ou 8h00 de travail, ils ont bien fait 8.”*

IIp6: Il mentionne un exemple d’abandon de projet: *“Dans une entreprise, ils voulaient aussi utiliser des montres connectées pour mesurer le nombre de pas par jour et puis les montres, elles avaient un système de géolocalisation... Et du coup, c'était évident que, à tout moment, avec ce système-là, ben l'employeur, si il avait accès aux données en temps-réel, il pouvait savoir qui se trouvait où, à quel moment. Et typiquement, c'est facile de pister, par exemple, le nombre de pauses prises dans la journée, la durée des pauses, où se trouvent les gens, etc, etc. Et juste, en les confrontant, finalement, ça avait été très rapidement abandonné. Alors que le… La raison initiale était plutôt louable sur le principe, mais il y avait tellement de risques de déviance que… que ça valait même pas la peine d'essayer.”*

**Section “Goal relevance / Limits for the acceptability of the device”**

IIp9: Il précise que *“le seul objet de cette chaise, ça devrait être la protection de la santé, et ça devrait être le seule et unique objet.”*

**Section ”Adverse Side Effects / side effects for employees”**

FGp21: Il détaille qu’*“il y aurait plein de façons de de faire en sorte que le système ne marche pas. Ou en tout cas marche mal.”*

IIp16: Elle souligne: *“Mais non parce que on peut pas mesurer … on peut pas appliquer de façon généralisée quelque chose à tous les individus !”*

IIp6: Il mentionne le risque principal de *“détournement clair de… l'utilité première de l'outil.”*

IIp7: Elle explique: *“En partant de ce principe-là [de santé et sécurité au travail], on est ouvert à accueillir toutes ces informations.  Mais après (...) ça va loin ! On place des capteurs chez les gens. (...) Après ils disent: (...) ‘c'est juste pour [vôtre bien-être]. Mais en fait (...) au fond, tout ce qui est derrière en fait, on le maîtrise pas.”*

IIp6: Il explique que entre *“deux collègues dans les mêmes conditions, il y en a un qui résiste mieux au stress, l'autre moins bien, tout d'un coup, on doit faire un choix en termes de poste de travail. Si l’employeur a accès à ce genre de données on va choisir logiquement la personne qui, en théorie, résiste mieux au stress.”*

IIp10: Elle souligne que le risque est *“d'augmenter la charge des employés si on voit que le niveau de stress il est assez bas dans l'entreprise. On dit « ah bon ben ils sont pas trop stressés, on va pouvoir augmenter le niveau de… de charge.”*

FGg24*: “Parce que moi (…) que la machine me dise: ‘fais une pause’, ça va pas vraiment m'aider à réduire mon stress, ça va être si ça se trouve encore pire quoi."*

IIp14: Il alerte sur le fait que “*finalement ici, l’entreprise s’immisce dans le fait que ces employés marchent ou pas, finalement, c'est… ça sort de la sphère professionnelle. Et l'entreprise commence à surveiller en fait le… le comportement de l'employé pendant mais aussi en dehors du travai1.”*

**Section ”Adverse Side Effects/ Side effects for companies and for the society”**

IIp6: Il souligne la relation de dépendance humain-machine: *“En plus, je trouve que c'est très (...) automatique et machine-dépendant alors que, le stress, [il] y a quand même un côté humain et émotionnel, et je suis pas sûr que de le gérer de cette façon-là, c'est vraiment le… qu’on réussisse à atteindre l'objectif escompté.”*

**Section “Role of employees / Freedom of Choice”**

IIp7: Elle souligne que *“le risque majeur que je vois, ce serait, effectivement (...) que certaines portes se ferment à des collaborateurs pour une raison de mauvaise posture”*.

IIp14: Il explique qu’*“[il] y a des gens qui sont peut-être pas très sportifs (...) des gens qui sont des personnes à mobilité réduite: ça les exclut de facto.”*

**Section “Role of employees / Information Processing”**

IIp8: Il souligne que *“du point de vue éthique [...les employés] vont [commencer à] se poser la question: ‘Mais pourquoi vous voulez nos donneées ? L'entreprise, elle veut nous fliquer!’”*

IIp14: Il souligne les limites de *“la technologie en fait, parce que souvent pour tous ces chaises de bureau on dit ‘si utilisée correctement, ça peut permettre d'éviter XYZ problèmes de santé’ mais tout le problème est là, c'est l'utilisation correcte et sans aide extérieure avec simplement un mode d'emploi. C'est très difficile de savoir si on se situe ou pas dans les… dans les cadres de cette utilisation correcte.”*

**Section “Role of employees / Employee’s implication”**

IIp1: Il explique: *“Même, disons même, je pense, s'il est.. si on dit aux gens ‘oui mais vous inquiétez pas, les données sont (...) Anonymisées’. Je crois que les gens n'y croient plus maintenant. Honnêtement parlant, c'est du vent. Même si c'est vrai, si l'entreprise fait tout ce qu'il faut, il y a un doute.”*

IIp1: Il développe que *“les gens dans les sociétés, je dirais suisses, français, (...) sont assez accoutumés maintenant à tout ce qui est technologie (...). Donc ils auront pas tellement peur de (...) ce genre de chose.”*

IIp17: Il précise que dans cette situation: *“Alors là je pose pas la question à mon employeur car j'ai pas envie de mal prendre mon employeur donc je lui fais confiance aussi attention à pas faire n'importe quoi avec mon ordinateur évidemment, mais je pense cette question elle se pose.”*

**Section “Data Process / Specific risks related to data flow***”*

IIp6: Il alerte sur le fait que le partage de données par groupe comporte des risques: *“[Il] y a quand même dans ma pratique, j'ai quand même été confronté à un certain nombre de fois à un intérêt (...) clairement inadéquat de la [des] ressources humaines ou management pour avoir accès à ces données collectives ou à des données collectives qui sont clairement des données de santé et des données médicales. Et (...) la raison de l'accès c'était clairement pas (...) la prévention, mais c'était vraiment du… du contrôle du contrôle des employés...*”

IIp6: Il s’interroge: *“Qui a accès aux données? BioDat? FitChairs? Le médecin du travail, l'entreprise, les ressources humaines? Enfin c'est pas, c'est pas clair.”*

IIp5: Il souligne : *“Ca me paraît louche que cette entreprise-là* [celle qui effectue l’analyse des données] *ne fasse rien de ces données.”*

IIp5: Il explique qu’il faut*“avoir un peu plus de transparence, un peu plus d'information sur la manière dont est faites l’analytics.”*

**Section “Data Process / Data Security”**

IIp14: Il soulighe que *“à la fin on a le rapport qui est envoyé aux ressources humaines, donc… alors après, même si c'est anonymisé, je pense que les ressources humaines doivent à peu près savoir qui a choisi de faire partie de ce programme ou pas.”*

**Section “Data Process / Lack of Trustworthy Actor”**

IIp6: Il souligne le rôle limité du médecin du travail: *“On n'est jamais un garde-fou obligatoire parce qu'on n'est jamais, quand il y a des projets, on n'est jamais mentionné comme étant une personne qui doit donner son avis obligatoirement quel que soit le type de projet, non. Parfois on est informé en amont parce que les gens se rendent compte que c'est un projet qui touche la santé ou des données médicales et on… les gens vont le présenter au service de santé ou aux médecins du travail, puis parfois pas du tout et puis on apprend quelques semaines ou quelques mois après l'implémentation du projet qu'il existe.”*

**Section “Vagueness / Unclear Employer’s Obligations”**

IIp9: Il souligne que *“le problème c'est qu'en Suisse on est dans un no man's land juridique sur la surveillance des employés. (...) Il y a peu de jurisprudence et il y a peu de choses.”*

**Section “Vagueness / Uncertainty about product’s adequacy”**

IIp6: Il précise: *“Sachant que évidemment, je suis pas du tout opposé au high-tech et au développement de ces technologies-là, mais parfois on parfois on développe d'abord la technologie et la récolte de données, puis après on se pose la question de est-ce que c'est pertinent ou pas ? Pis comme on a développé la chose, ben au niveau marketing, on doit vendre le produit, puis après on essaie de bricoler une utilité à ce qu'on a développé et on prétend que. Et dans ce scénario-là, j'ai l'impression qu'on est plutôt de ce côté-là de la chose...”*

**Section “Follow-up / Role of Employees”**

IIp10: Selon lui, *“si c'est discuté avec les employés et pis que il y a je sais pas, une charte, un truc qui… qui qui donne les garanties que… voilà, de nouveau, on peut (...) jamais exclure des dérives ! Mais (...) c'est ce ratio bénéfice-risque: voilà si les garanties sont suffisantes, on peut dire ‘oui bon bah voilà.’”*

IIp16: Elle souligne que *“ça change énormément parce, on perd le libre arbitre. On peut plus dire ‘non, je m'oppose!’”*

IIp13: Il explique: *“J'ai l'impression qu’aujourd'hui ils se rendent pas compte, (...) comment déjà ils partagent déjà énormément des données privées personnelles de leur côté ! (...)  Et puis par contre, dès que il y a un outil spécifique qui est implémenté, c'est là tout de suite qu’ils viennent avec toutes la sensibilité par rapport à leurs données, qui est juste une contradiction de principe. Mais c'est le grand problème aujourd'hui.”*

**Section “Follow-up / Side effects”**

IIp20: Elle explique: “*Le principal effet secondaire du Big data qu'on n'a pas encore bien ressenti, (...) c'est celui de la…de l'effacement de la singularité. Et ça, c'est quelque chose qui va être, dont il est difficile de juger  des conséquences.”*

**Section “Follow-up / Competent authorities”**

FGp22: Il explique: “*Si c'était [l‘organisation]* Promotion Santé *qui avait ce genre d'offre [de déploiement d’objets connectés à des fins de santé sécurité au travail] pour de grandes entreprises, moi (...) je ferais beaucoup plus confiance parce qu[‘elle] doit aussi garantir que (...) c'est un projet*
